# The Role of Self-Management in Pessary Therapy for Pelvic Organ Prolapse—A retrospective cohort study

**DOI:** 10.1007/s00192-024-05864-7

**Published:** 2024-07-24

**Authors:** Evy Paulussen, Renée Börger, Hugo van Eijndhoven, Marian Engberts, Pieternel Steures, Mirjam Weemhoff

**Affiliations:** 1 Faculty of Health, Medicine & Life Sciences, Maastricht University, Maastricht, The Netherlands; 2 Department of Gynaecology, Zuyderland Medical Centre, Heerlen, The Netherlands; 3 Department of Gynaecology, Isala Medical Centre, Zwolle, The Netherlands; 4 Department of Gynaecology, Jeroen Bosch Medical Centre, ’s-Hertogenbosch, The Netherlands; 5 GROW Maastricht University, Maastricht, The Netherlands

**Keywords:** Conservative treatment, Pelvic organ prolapse, Pessary, Self-management, Urogynaecology

## Abstract

**Introduction and Hypothesis:**

This study investigated pessary self-management (PSM). The primary outcome was how often PSM was taught to patients with pelvic organ prolapse (POP). Secondary outcomes were associations of PSM with treatment continuation, side effects, changing to surgery, and number of doctor consultations in the first year after treatment initiation compared with clinical management (CM).

**Methods:**

A retrospective cohort study was conducted in 300 patients visiting three Dutch medical centres in 2019, and receiving a pessary for POP. The *t* test, Chi-squared test and logistic regression were performed to compare PSM with CM and to identify factors associated with treatment continuation.

**Results:**

A total of 35% of patients received PSM instructions, of which 92% were able to perform PSM successfully. Treatment was continued by 83% of patients practicing PSM and 75% of patients having CM (*p* = 0.16), side effects occurred in 26% and 39% respectively (*p* = 0.18). Pain or discomfort was associated with treatment discontinuation (*p* < 0.01). In a subgroup analysis of patients who had a pessary suitable for PSM, treatment continuation was significantly higher in the PSM group (97%) than in the CM group (74%; *p* < 0.01).

**Conclusions:**

Pessary self-management was only taught to 35% of patients who received a pessary, although the ability to perform PSM was high (92%). Treatment discontinuation was significantly lower in the PSM subgroup, when assessing the subgroup of patients using a pessary suitable for PSM. The large number of patients using a pessary suitable for PSM in the CM group implies that there is a lot to gain by promoting PSM.

## Introduction

Pelvic organ prolapse (POP) occurs in 40% of women aged 45 years and older. POP can be treated using pelvic floor physical therapy, a pessary or surgery. Pessary treatment and prolapse surgery are both equally effective methods for addressing prolapse complaints. Individual preferences play a role in the choice of treatment [1]. Approximately 50% of women opt for surgery, whereas the other half choose a pessary [2, 3]. However, pessary therapy is often discontinued because of side effects such as vaginal discharge, vaginal blood loss due to lacerations, discomfort, and problems with sexual intercourse [4]. Traditionally, pessary treatment is accompanied by clinical management (CM), which means visiting a general practitioner or gynaecologist a few times a year to remove, clean, and replace the pessary to prevent complications.

With aging contributing to an unsustainable growth in health care, it is becoming increasingly important to promote self-management to decrease the number of doctor consultations. PSM has the potential to reduce health care pressure and costs [5, 6]. There is a large variety of types of pessaries, some of which are only suitable with PSM (e.g. cube pessary), some are more suitable for PSM (ring pessaries) and some are less suitable for PSM (e.g. Gellhorn or donut) [7]. A UK study reports a success rate up to 73% for PSM continuation after 6 months in patients who were willing to learn PSM [6]. In two Dutch studies, PSM was offered to 184 and 163 patients, of whom 34% and 45% respectively continued self-management after 12 months [8, 9]. Furthermore, PSM has the potential to enhance patient autonomy, to prevent (worsening of) side effects and to minimise the need for surgical intervention. However, evidence of these positive potentials in self-management is lacking.

The aim of this retrospective multicentre cohort study was to determine how often pessary self-management was taught and succeeded in daily practice. Treatment continuation, number of side effects, crossover to surgical intervention and number of consultations in the first 12 months after pessary treatment initiation were compared between patients performing PSM and patients receiving CM. Furthermore, factors that are associated with discontinuation of pessary therapy were identified.

## Materials and Methods

This descriptive retrospective cohort study, approved by the Medical Ethical Committee (METCZ20220053), compared PSM with CM in patients with POP in three secondary care medical centres in the Netherlands.

### Subjects

Patients visiting a gynaecologist in the participating centres with symptoms of POP, and who preferred to try pessary therapy, were eligible for this study. In each participating centre the first 100 consecutive patients were included if their trajectory of care started in 2019, the insurance code for POP was applied and the transaction code “fitting a pessary” was registered. Pessaries were inserted by gynaecologists specialised in pelvic floor disorders. When a patient with prolapse opted for pessary therapy, initially an open ring pessary was placed. If there was descensus uteri or insufficient support with an open ring, a ring with support was used. If these pessaries did not remain in place owing to insufficient pelvic floor support or a wide hiatus, a filling pessary was chosen. For sexually active women, a cube pessary was preferred, whereas a Gellhorn pessary was often used for those who were not sexually active. If the patient had previously used an open ring or ring with support placed by their general practitioner and was referred because of its failure, a filling pessary was directly chosen. Self-management was taught after successful fitting of a pessary. Successful fitting was stated when the pessary remained properly in place and the patient wished to continue pessary therapy.

The patient was then either referred back to the general practitioner for further follow-up or follow-up took place in the medical centre every 4–6 months. The gynaecologist considered that PSM was feasible in her daily experience. There was no consensus on how to teach PSM. For the cube pessary, PSM was taught immediately after the initial placement as daily removal is essential. Table 1 showed how PSM was taught in the three medical centres in 2019.

**Table 1:** Pessary self-management (*PSM*) instruction method in three Dutch medical centres in 2019

	Medical centre 1	Medical centre 2	Medical centre 3
Information video or leaflet before learning PSM?	No	No	Yes, leaflet
Separate consult?	No	No	Usually during the first consultation, sometimes in a second consult, depending on the woman’s wish
Teaching PSM?	The gynaecologist demonstrated how to insert and remove the pessary. Subsequently, the patient attempted to insert and remove the pessary herself with the assistance of the gynaecologist. Finally, she inserted and removed the pessary on her own	The gynaecologist demonstrated how to insert and remove the pessary. Subsequently, the patient attempted to insert the pessary herself, with one leg elevated on a chair or toilet	The gynaecologist demonstrated how to insert and remove the pessary. Subsequently, the patient attempted to insert the pessary herself, with one leg elevated on a chair or toilet

To demonstrate a 20% difference between the PSM and the CM group regarding treatment continuation after 12 months with a power of 80%, a minimum of 52 women needed to be included in each group. Gynaecologists working in the involved medical centres estimated that 20% of the patients receiving pessary treatment in 2019 were taught self-management. Therefore, a total of 300 women had to be included to make sure the research population included 60 women performing PSM.

### Data Collection

Primary outcome of this study was how often pessary self-management (PSM) was taught to patients with POP. Secondary outcomes were treatment continuation, side effects, changing to surgery, and number of doctor consultations in the first year after treatment initiation compared with CM.

The following data were extracted from the electronic health records of the 300 enrolled patients: age, body mass index (BMI), previous hysterectomy, prolapse surgery or pessary use, POP-Q items and stage, most dominant type of POP, whether PSM instructions were given, whether patients were able to perform PSM, the manner and location of follow-up, the type and size of the pessary, the side effects and other negative effects or reasons to discontinue pessary treatment, treatment continuation at 12 months after fitting a pessary, surgery (planned) within 12 months after fitting a pessary and number of consultations in the first 12 months after fitting a pessary [10].

### Data Analysis

All data were entered and analysed in the Statistical Package for the Social Sciences (SPSS statistics for Windows, version 26.0; IBM, Armonk, NY, USA). Distribution of data was tested for continuous variables. Data were presented using the mean (SD) for equally distributed variables and the median (minimum/maximum) for non-equally distributed variables. Differences in baseline characteristics were analysed for the PSM and CM groups. Furthermore, treatment continuation, negative effects, crossover to surgery and number of doctor consultations 12 months after treatment initiation were analysed by using Student's *t* test for continuous variables and Chi-squared test for categorical variables. A subgroup analysis was performed to compare PSM and CM in patients using a pessary that was suitable for PSM, excluding the cube (PSM-only option), Gellhorn and other filling pessaries.

To investigate factors associated with pessary therapy continuation at 12 months, binary logistic regression analysis was performed. First, the continuation and discontinuation groups were compared using univariate logistic regression to detect a possible association per factor. Second, all factors with *p* < 0.05 were included in the multivariate logistic regression analysis to assess the independent factors associated with pessary treatment continuation. In all analyses, *p* values < 0.05 were considered statistically significant.

When the medical record did not state any information regarding PSM instructions, previous prolapse surgery, previous hysterectomy or previous pessary use, it was assumed that these events did not occur. When there was no notification about side effects or cross-over to surgery, these factors were assumed to be negative. The last notification of pessary use was extracted for analysis, assuming that the patient continued PSM. Missing values for the location of follow-up and the continuous variables BMI and pessary size were not imputed. This is reflected in the denominator of the subjects to be described.

## Results

**Fig. 1:**
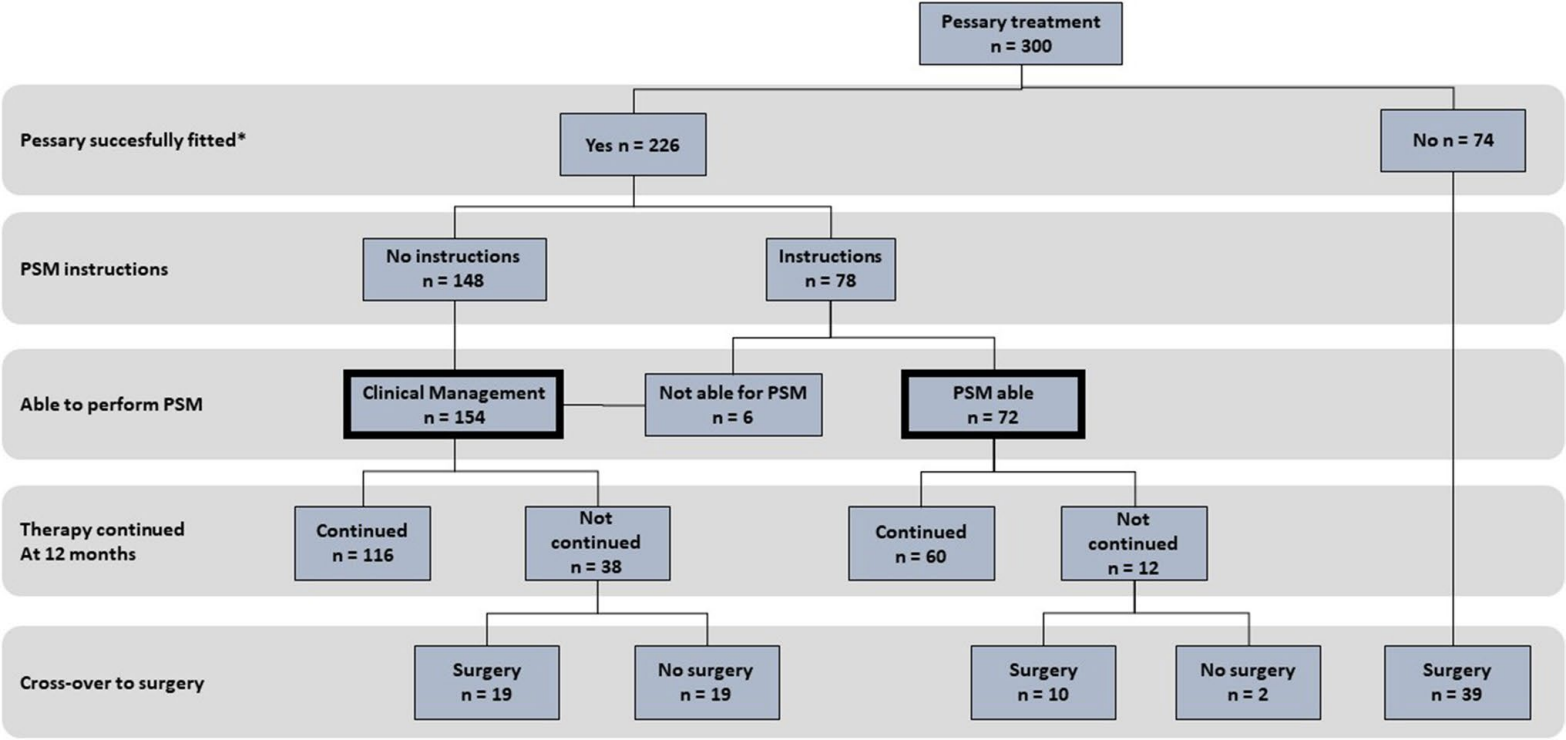
Patient flow. *Successful fitting was stated when the pessary remained properly in place and the patient wished to continue pessary therapy. *PSM* pessary self-management

The study was carried out between November 2022 and July 2023. A total of 300 patients, visiting the medical centre in 2019, were included (Fig. 1). A pessary was fitted successfully in 226 patients (75%). Seventy-four patients (25%) lost their pessary after fitting and discontinued pessary therapy for this reason. These patients, of whom 24 were in the PSM group and 50 were in the CM group, were excluded from further analysis.

Instructions on PSM were given to 78 out of 226 patients (35%), of whom 72 women were able to perform PSM (92%). Six women failed to self-manage; these women instead received CM. The main reason for not being able to perform PSM was the inability to reach the pessary (*n* = 5) and in 1 woman self-management failed owing to comorbidity. In total, 154 women received CM (68%). Of the 78 women who received PSM instructions, 30 (38%) were fitted with a cube pessary.

Table 2 shows the baseline characteristics of the patients in the two groups. Age and pessary size were significantly lower in the PSM group than in the CM group (58 vs 69 years, *p* < 0.01 and 56 vs 71 mm, *p* < 0.01). No significant differences were found for BMI, medical history, and the most dominant type and stage of prolapse.

**Table 2:** Patient characteristics and treatment of patients in whom a pessary was successfully fitted, pessary self-management vs clinical management

	Self-management (*n* = 72)	Clinical management (*n* = 154)	*p*
Characteristics			
Age, mean (SD)	58 (13)	69 (12)	** < 0.01**
BMI, mean (SD)	25.9 (3.5) (*n* = 53)	26.3 (4.1) (*n* = 127)	0.47
Medical history, *n* (%)			
Hysterectomy	9 (13)	21 (14)	0.77
Previous prolapse surgery	6 (8)	15 (10)	0.70
Previous pessary use	28 (39)	49 (32)	0.35
Most dominant type, *n* (%)			
Cystocele	44 (61)	102 (66)	0.35
Rectocele	9 (13)	13 (8)	0.36
Descensus uteri	7 (10)	23 (15)	0.48
Combination	12 (17)	16 (10)	0.20
Most dominant stage, *n* (%)			
Mild	55 (76)	110 (71)	(ref)
1	6 (8)	5 (3)	
2	47 (65)	89 (58%)	
Severe	17 (24)	53 (34)	0.16
3	17 (24)	50 (32)	
4	0 (0)	3 (2)	
Pessary, *n* (%)			
Only suitable for PSM			n/a
Cube	30 (42)	0 (0)	
Suitable for PSM	36 (50)	123 (80)	n/a
Ring (n, %)	24 (33)	68 ()	
Ring with support	11 (15)	43 (28)	
Knob	1 (1)	12 (8)	
Less suitable for PSM	6 (8)	31 (20)	n/a
Gellhorn	0 (0)	10 (7)	
Falk	6 (8)	15 (10)	
Donut	0 (0)	1 (1)	
Shaatz	0 (0)	5 (3)	
Size (mm), mean (SD)	56.4 (17.7) (*n* = 69)	70.5 (8.9) (*n* = 200)	** < 0.01**

Independent samples *t* test for continues variables, Chi-squared test for categorical variables

*PSM* pessary self-management, *n/a* not applicable

### Pessary Self-Management Versus Clinical Management

In the PSM group, 26% of the patients (19 out of 72) had one or more side effects, compared with 39% (60 out of 154) in the CM group (*p* 0.18). No statistical differences were found between the PSM and CM groups in the occurrence of problematic vaginal discharge, decubitus-related symptoms (erythema, ulceration, vaginal blood loss), pain or discomfort, or problems with intercourse (Table 3).

**Table 3:** Negative effects and treatment results of patients for whom a pessary was successfully fitted, pessary self-management vs clinical management

	Self-management (*n* = 72)	Clinical management (*n* = 154)	*p*
Negative effects, *n* (%)			
> 1 negative effect	19 (26)	60 (39)	0.18
Vaginal discharge	4 (6)	12 (8)	0.54
Decubitus related	8 (11)	21 (14)	0.60
Pain/discomfort	7 (10)	23 (15)	0.28
Intercourse related	2 (3)	8 (5)	0.41
Results, *n* (%)			
Pessary therapy continued	60 (83)^a^	116 (75)^b^	0.16
Cross-over to surgery	10 (14)	19 (12)	0.79
Number of consultations, mean (SD)	3.0 (1.8)	3.3 (1.5)	0.25

*SUI* stress urinary incontinence

^a^Of which 22 in whom the last observation was carried forward (31%)

^b^Of which 40 in whom the last observation was carried forward (26%)

Pessary treatment was continued at 12 months in 83% of the women (60 out of 72) who performed PSM and in 75% of the women (116 out of 154) in the CM group (*p* = 0.16). In the 1st year the PSM and CM groups had an average of 3.0 (± 1.8) and 3.3 (± 1.5) doctor consultations respectively (*p* = 0.25). Cross-over to surgery occurred in 14% of the PSM group (10 out of 72) and 12% of the CM group (19 out of 154) (*p* = 0.79; Table 3).

### Subgroup Analysis in Women with Pessaries Suitable for PSM

When analysing only patients with a pessary that is suitable for PSM and excluding the cube (only suitable for PSM), Gellhorn, donut and Shaatz pessary (not suitable for PSM), 36 patients remained in the PSM group and 123 patients in the CM group. In this subgroup analysis, age was significantly lower in the PSM group than in the CM group, 56 and 68 years respectively (*p* < 0.01). No statistically significant differences were observed in BMI, medical history, type and stage of prolapse and type of pessary (ring, ring with support or knob). In patients with a pessary suitable for PSM the treatment continuation was significantly higher in the PSM group than in the CM group (97% vs 74%, *p* < 0.01; Table 4).

**Table 4:** Negative effects and treatment results of patients for whom a pessary suitable for pessary self-management (*PSM*) was successfully fitted, pessary self-management vs clinical management

	Self-management (*n* = 36)	Clinical management (*n* = 123)	*p*
Negative effects, *n* (%)			
> 1 negative effect	10 (28)	46 (37)	0.29
Vaginal discharge	3 (8)	9 (7)	0.84
Decubitus related	5 (14)	12 (10)	0.48
Pain/discomfort	1 (3)	16 (13)	0.08
Intercourse related	2 (6)	6 (5)	0.87
Results, *n* (%)			
Pessary therapy continued	35^a^ (97)	91^b^ (74)	** < 0.01**
Cross-over to surgery	1 (3)	15 (12)	0.10
Number of consultations, mean (SD)	3.2 (1.8)	3.1 (1.4)	0.67

^a^Of which 13 were imputed (36%)

^b^Of which 36 were imputed (30%)

### Factors Associated with Treatment Continuation

The univariate analysis showed that previous hysterectomy (OR 0.43, 95% CI 0.19–0.96, *p* 0.04), rectocele type prolapse (OR 0.29, 95% CI 0.12–0.73, *p* < 0.01), and having one or more side effects (OR 0.21, 95C% CI 0.11–0.40, *p* < 0.01), such as pain or discomfort (OR 0.07, 95% CI 0.03–0.18, *p* < 0.01), were associated with higher discontinuation of pessary treatment rates at 12 months. Uterine descent (OR 4.54, 95% CI 1.04–19.77, *p* 0.04) and larger pessary size (OR 1.03, 95% CI 1.00–1.05, *p* 0.03) were associated with higher treatment continuation at 12 months.

In the multivariate analysis, pain or discomfort remained the only significant independent variable associated with the discontinuation of pessary treatment (OR 0.13, 95% CI 0.04–0.39, *p* < 0.01; Table 5).

**Table 5:** Univariate and multivariate binary logistic regression of patients for whom a pessary was successfully fitted, continued vs discontinued at 12 months

	Continued (*n* = 176*)	Discontinued (*n* = 50)	Univariate			Multivariate		
Characteristics			OR	95% CI	*p*	OR	95% CI	*p*
Age, mean (SD)	66.4 (13.5)	63.3 (13.9)	1.02	0.99–1.04	0.16			
BMI, mean (SD)	26.0 (3.8) (*n* = 134)	26.7 (4.4) (*n* = 46)	0.96	0.88–1.04	0.30			
Medical history, *n* (%)								
Hysterectomy	19 (11)	11 (22)	0.43	0.19–0.96	**0.04**	0.50	0.18–1.38	0.18
Previous prolapse surgery	16 (9)	5 (10)	0.90	0.31–2.59	0.85			
Previous pessary use	67 (38)	10 (20)	2.50	1.15–5.24	0.02			
Most dominant type, *n* (%)								
Cystocele	116 (66)	30 (60)	1.29	0.68–2.46	0.44			
Rectocele	12 (7)	10 (20)	0.29	0.12–0.73	** < 0.01**	0.48	0.16–1.48	0.20
Descensus uteri	28 (16)	2 (4)	4.54	1.04–19.77	**0.04**	4.01	0.81–20.00	0.09
Combination	20 (11)	8 (16)	0.67	0.28–1.64	0.38			
Most dominant stage, *n* (%)								
Mild	119 (68)	37 (74)	1 (ref)					
Severe	57 (32)	13 (26)	1.36	0.67–2.76	0.39			
Pessary, *n* (%)								
Only PSM suitable	19 (11)	11 (22)	0.45	0.20–1.04	0.06			
PSM suitable	126 (72)	33 (66)	1 (ref)					
Less suitable for PSM	31 (18)	6 (12)	1.35	0.52–3.51	0.54			
Size, mm, mean (SD)	67.2 (13.4) (*n* = 171)	62.1 (15.6) (*n* = 48)	1.03	1.00–1.05	**0.03**	1.02	0.99–1.04	0.28
Negative effects, *n* (%)								
> 1 negative effect	47 (27)	32 (64)	0.21	0.11–0.40	** < 0.01**	1.97	0.82–4.72	0.13
Vaginal discharge	12 (7)	4 (8)	0.84	0.26–2.73	0.77			
Decubitus related	23 (13)	6 (12)	1.10	0.42–2.88	0.84			
Pain/discomfort	9 (5)	21 (42)	0.07	0.03–0.18	** < 0.01**	0.13	0.04–0.39	** < 0.01**
Intercourse related	6 (3)	4 (8)	0.41	0.11–1.50	0.18			
PSM, *n* (%)	60 (34)	12 (24)	1.68	0.82–3.45	0.16			

*PSM* pessary self-management

^a^Of which 62 in whom the last observation carried forward (35%)

## Discussion

In this retrospective cohort study in 300 patients with POP, a pessary was fitted successfully in 226 patients (75%). PSM instructions were given to 35% of patients. Among them, 92% were able to perform PSM. Patients who performed PSM had 8% higher treatment continuation and 13% fewer side effects than the CM group; these differences were not statistically significant. In the subgroup analysis of women using a pessary that was suitable for PSM the treatment continuation was significantly higher in the PSM group (97%) than in the CM group (74%).

Pessary fitting was successful in 75% of patients, which corresponds to several studies reporting pessary-fitting success rates at 77% and 71% [3, 11]. The prevalence of PSM instructions and ability to perform PSM was also in line with previous research [11–14].

In the CM group 80% of the patients had a pessary that was suitable for PSM (e.g. ring, ring with support or ring with knob). This implies that a considerable number of regular visits can be turned into self-management, shifting from follow-up consultation by a gynaecologist or general practitioner to self-care by patients themselves. Therefore, it is expected that self-management will result in a reduction of demand and costs in health care systems. However, our findings showed no difference between doctor consultations in PSM and CM groups in the 1st year. An explanation for this finding is that consultations for fitting a pessary, for giving the instructions of self-management and for evaluation of the therapy take place in the 1st year after starting the pessary treatment. Therefore, a longer time frame is needed to detect a difference in demand and costs. Our hypothesis in terms of health care usage and costs are in line with the findings of Kearney and Brown. They estimated that health care costs were ten times higher if pessary treatment was performed with CM by doctors in comparison with PSM by patients themselves [6]. Future studies focusing on cost-effectiveness should include a follow-up duration of more than 1 year.

Treatment continuation in the subgroup of patients using a pessary that is suitable for PSM, was significantly (p < 0,01) higher in the PSM group (97%) than in the CM group (74%). The continuation rate is in line with findings of Ma et al., who reported treatment continuation 1 year after successful pessary fitting in 88% of women who were offered PSM. At 5 years, treatment continuation was reported in 80% for patients performing PSM and in 59% for patients who were unable to perform PSM [11]. Some studies found lower success rates, 73% at 6 months [6], and 34–45% at 12 months after PSM treatment initiation [8, 9], but these studies incorporated patients for whom pessary fitting as such was unsuccessful in their analysis; this does not reflect the outcome of PSM specifically.

Cross-over to surgery was similar in the PSM and CM groups (14% and 12%). Although there was a difference in pessary treatment continuation in the subgroup analysis of patients using a pessary suitable for PSM, cross-over to surgery did not differ significantly (3% and 12%). A Dutch study reported the rate of cross-over to surgery to be 24%, which is higher than that we found in our study [3]. This outcome can be attributed to the exclusion of patients with unsuccessful pessary fitting in our results.

The prevalence of side effects in pessary treatment was similar to those found by Van der Vaart et al. [3]. The number of patients with one or more side effects differed 13% between the PSM and CM groups, but in our sample size this difference was not significant.

Age was significantly lower in the PSM group than in the CM group. Several studies reported similar results, which can be explained by generational attitudes of general practitioners and gynaecologists that younger women are often better equipped to perform PSM. It is possible that these beliefs of health care providers influenced which patients were initially offered training in PSM [8, 13–15].

Furthermore, pessary size significantly differed between groups. This difference can be explained by the fact that all patients with cube pessaries were in the PSM group, and these pessaries regularly had a smaller diameter than other pessaries. After excluding the cube pessary from the statistical analyses a significant difference in pessary size between the two groups was no longer found.

The multivariable regression analysis of factors that were associated with discontinuation of pessary treatment after 12 months showed that only pain or discomfort in pessary treatment was predictive of discontinuation. In the 5-year follow-up study of Ma et al. PSM appeared to be predictive of continuation of pessary treatment [11]. In the entire study population our results showed no association between PSM and continuation of treatment. In the subgroup of patients with a pessary suitable for PSM, we did find a higher continuation rate in the PSM group than in the CM group.

In several studies older age appeared to be positively associated with pessary treatment continuation, suggesting that conservative treatment might be preferred by older patients and/or their physicians [9, 16–19]. However, in our study we did not find a significant association between age and continued pessary use.

A strength of our study is that it provides an accurate representation of current daily pessary care in the Netherlands because of the multicentre design and inclusion of 300 consecutive patients receiving a pessary for POP during their first visit to the gynaecologist, without any other criteria. Current literature focuses largely on the success of pessary therapy in general and not primarily on how often counselling on PSM takes place and the results of training in PSM.

A limitation of this study was that the retrospective data collection from electronic health records led to incomplete information, and assumptions had to be made in cases of missing data (longitudinal imputation of last observation carried forward). These assumptions made this study prone to information or ascertainment bias. As one of our primary outcomes measured discontinuation of pessary treatment, this longitudinal imputation was performed to maximise data availability rather than excluding 62 patients (22 in the PSM group and 40 in the CM group). This was mainly the case for patients who were referred back to the general practitioner for follow-up, and for patients who performed PSM and would only come back to the gynaecologist on indication. However, it is possible that some patients discontinued pessary treatment without consulting their gynaecologist.

Second, it should be noted that PSM instructions are offered to a selected group of patients in current medical practice. The decision-making process of physicians offering PSM to individual patients, those who were expected to perform PSM successfully, was beyond the scope of this study. The proportion of patients able to perform PSM would likely be lower, if PSM instructions were offered to all women.

At last, the limited sample size and relatively short duration of follow-up limits us to making strong conclusive statements about differences between PSM and CM in the total population. Therefore, we recommend a prospective cohort or randomised controlled trial design with a longer duration of follow-up and a larger sample size for future studies.

## Conclusion

Pessary self-management was taught to only 35% of patients for whom a pessary was successfully fitted. Among these patients, 92% were able to perform PSM. Treatment continuation in the subgroup of patients using a pessary that was suitable for PSM was significantly higher in patients performing PSM than in those visiting a gynaecologist or general practitioner for CM. Pain or discomfort was associated with pessary therapy discontinuation at 12 months.

The large number of patients using a pessary suitable for PSM in the CM group implies that there is much to be gained by promoting PSM. We therefore advise clinicians to provide patients with a pessary suitable for PSM with appropriate instructions to promote self-management and improve the results of pessary treatment.

## Data Availability

The data that support the findings of this study are available from the corresponding author, upon reasonable request.

## References

[1] Coolen AWM, Troost S, Mol BWJ, Roovers JPWR, Bongers MY. Primary treatment of pelvic organ prolapse: pessary use versus prolapse surgery. Int Urogynecol J. 2018;29(1):99–107. 10.1007/s00192-017-3372-x. 28600758 10.1007/s00192-017-3372-x PMC5754400

[2] Kapoor DS, Thakar R, Sultan AH, Oliver R. Conservative versus surgical management of prolapse: what dictates patient choice? Int Urogynecol J Pelvic Floor Dysfunct. 2009;20(10):1157–61. 10.1007/s00192-009-0930-x. 19543676 10.1007/s00192-009-0930-x

[3] Van der Vaart LR, Vollebregt A, Milani AL, Lagro-Janssen AL, Duijnhoven RG, Roovers JP, Van der Vaart CH. Pessary or surgery for a symptomatic pelvic organ prolapse: the PEOPLE study, a multicentre prospective cohort study. BJOG. 2022;129(5):820–9. 10.1111/1471-0528.16950. 34559932 10.1111/1471-0528.16950 PMC9298049

[4] Sarma S, Ying T, Moore KH. Long-term vaginal ring pessary use: discontinuation rates and adverse events. BJOG. 2009;116(13):1715–21. 10.1111/j.1471-0528.2009.02380.x. 19906018 10.1111/j.1471-0528.2009.02380.x

[5] Bugge C, Dembinsky M, Kearney R, Hagen S. Does self-management of vaginal pessaries improve care for women with pelvic organ prolapse? BMJ. 2021;19(372):n310. 10.1136/bmj.n310. 10.1136/bmj.n310 33608314

[6] Kearney R, Brown C. Self-management of vaginal pessaries for pelvic organ prolapse. BMJ Qual Improv Rep. 2014;3(1):u206180.w2533. 10.1136/bmjquality.u206180.w2533. 27493737 10.1136/bmjquality.u206180.w2533 PMC4949618

[7] Nemeth Z, Nagy S, Ott J. The cube pessary: an underestimated treatment option for pelvic organ prolapse? Subjective 1-year outcomes. Int Urogynecol J. 2013;24(10):1695–701. 10.1007/s00192-013-2093-z. 23579291 10.1007/s00192-013-2093-z

[8] Thys SD, Hakvoort RA, Asseler J, Milani AL, Vollebregt A, Roovers JP. Effect of pessary cleaning and optimal time interval for follow-up: a prospective cohort study. Int Urogynecol J. 2020;31(8):1567–74. 10.1007/s00192-019-04200-8. 31907565 10.1007/s00192-019-04200-8 PMC7363720

[9] Thys S, Hakvoort R, Milani A, Roovers JP, Vollebregt A. Can we predict continued pessary use as primary treatment in women with symptomatic pelvic organ prolapse (POP)? A prospective cohort study. Int Urogynecol J. 2021;32(8):2159–67. 10.1007/s00192-021-04817-8. 34002267 10.1007/s00192-021-04817-8

[10] Persu C, Chapple CR, Cauni V, Gutue S, Geavlete P. Pelvic Organ Prolapse Quantification System (POP-Q)—a new era in pelvic prolapse staging. J Med Life. 2011;4(1):75–81. 21505577 PMC3056425

[11] Ma C, Zhou Y, Kang J, et al. Vaginal pessary treatment in women with symptomatic pelvic organ prolapse: a long-term prospective study. Menopause. 2021;28(5):538–45. 10.1097/GME.0000000000001751. 33625108 10.1097/GME.0000000000001751

[12] Ramsay S, Bouchard F, Tu LM. Long term outcomes of pessary use in women with pelvic organ prolapse. Neurourol Urodyn. 2011;30(6):1105–6.

[13] Holubyeva A, Rimpel K, Blakey-Cheung S, Finamore PS, O’Shaughnessy DL. Rates of pessary self-care and the characteristics of patients who perform it. Female Pelvic Med Reconstr Surg. 2021;27(3):214–6. 10.1097/SPV.0000000000001013. 33620907 10.1097/SPV.0000000000001013

[14] Dwyer L, Dowding D, Kearney R. What is known from the existing literature about self-management of pessaries for pelvic organ prolapse? A scoping review. BMJ Open. 2022;12(7):e060223. 10.1136/bmjopen-2021-060223. 35851026 10.1136/bmjopen-2021-060223 PMC9297214

[15] Storey S, Aston M, Price S, Irving L, Hemmens E. Women’s experiences with vaginal pessary use. J Adv Nurs. 2009;65(11):2350–7. 10.1111/j.1365-2648.2009.05095.x. 19832750 10.1111/j.1365-2648.2009.05095.x

[16] Heit M, Rosenquist C, Culligan P, Graham C, Murphy M, Shott S. Predicting treatment choice for patients with pelvic organ prolapse. Obstet Gynecol. 2003;101(6):1279–84. 10.1016/s0029-7844(03)00359-4. 12798537 10.1016/s0029-7844(03)00359-4

[17] Oh S, Namkung HR, Yoon HY, Lee SY, Jeon MJ. Factors associated with unsuccessful pessary fitting and reasons for discontinuation in Korean women with pelvic organ prolapse. Obstet Gynecol Sci. 2022;65(1):94–9. 10.5468/ogs.21232. 34897264 10.5468/ogs.21232 PMC8784938

[18] Cheung RYK, Lee LLL, Chung TKH, Chan SSC. Predictors for dislodgment of vaginal pessary within one year in women with pelvic organ prolapse. Maturitas. 2018;108:53–7. 10.1016/j.maturitas.2017.11.008. 29290215 10.1016/j.maturitas.2017.11.008

[19] Manzini C, Morsinkhof LM, van der Vaart CH, Withagen MIJ, Grob ATM. Parameters associated with unsuccessful pessary fitting for pelvic organ prolapse up to three months follow-up: a systematic review and meta-analysis. Int Urogynecol J. 2022;33(7):1719–63. 10.1007/s00192-021-05015-2. 35037973 10.1007/s00192-021-05015-2 PMC9270314

